# Barriers and facilitators to palliative care for patients with non-curable cancer in Colombia: perspectives of allied health and social care professionals

**DOI:** 10.1186/s12904-023-01267-5

**Published:** 2023-10-06

**Authors:** Cindy V. Mendieta, Esther de Vries, Maria Elizabeth Gomez-Neva, Angela Maria Muñoz-Escudero, Jose Andrés Calvache, Tracey McConnell

**Affiliations:** 1 PhD Program in Clinical Epidemiology, Department of Clinical Epidemiology and Biostatistics, Faculty of Medicine, Pontificia Universidad Javeriana, Bogota, Colombia; 2 Department of Nutrition and Biochemistry, Faculty of Sciences, Pontificia Universidad Javeriana, Bogotá, Colombia; 3 Department of Clinical Epidemiology and Biostatistics, Faculty of Medicine, Pontificia Universidad Javeriana, Bogota, Colombia; 4 Department of Nursing Clinical, Faculty of Nursing, Pontificia Universidad Javeriana, Bogota, Colombia; 5 Department of Nursing, Faculty of Health Sciences, Universidad del Cauca, Popayán, Colombia; 6 Department of Anesthesiology, Faculty of Health Sciences, Universidad del Cauca, Popayán, Colombia; 7 Department of Anesthesiology, Erasmus University Medical Center, Rotterdam, The Netherlands; 8 Marie Curie Hospice Belfast, Belfast, UK; 9 Queen’s University Belfast School of Nursing and Midwifery, Belfast, UK

**Keywords:** Palliative care, cancer, Allied health and social care professionals, Barriers, Facilitators

## Abstract

**Background:**

Palliative care aims to improve the quality of life of people with life-limiting illness and their families by addressing physical, psychological, social and spiritual suffering. Allied Health and Social Care Professionals (AHSCP) are key to delivering comprehensive, high quality palliative care. In recent years, Colombia has developed changes in the legal, and regulatory framework for access to palliative care but barriers and facilitators to palliative care for patients with non-curable cancer have not been explored from the perspective of AHSCP.

**Method:**

This study aims to address this knowledge gap in two cities in Colombia: one in a medium-sized city in a rural area (Popayan) and one in a highly urbanized area (Bogota). Two focus groups with AHSCP were conducted using the World Cafe method, and a subsequent thematic analysis was performed to establish the main barriers and facilitators.

**Results:**

A wide range of 18 AHSCPs attended the two World Cafe groups in Popayan and Bogota. As a result of this iterative process, we established five thematic areas: (i) Humanizing care, (ii) Normalizing palliative care: referral at the time of diagnosis, (iii) Misunderstandings related to palliative care, (iv) Barriers within the health system, and (v) Geographic barriers.

**Conclusion:**

This study provided the perspectives of AHSCPs in Colombia in relation to barriers and facilitators in the framework of comprehensive palliative care attention. Participants identified misconceptions about palliative care, which are explained by the lack of inclusion of this area in the educational programs of health professionals and AHSCPs, along with the limited supply and access to palliative care, especially in rural areas.

**Supplementary Information:**

The online version contains supplementary material available at 10.1186/s12904-023-01267-5.

## Background

According to the World Health Organization (WHO), access to Palliative Care (PC) is a human right, and must be available for all adults, children with life-limiting illness and their families to prevent and relieve physical, psychological, social and spiritual suffering [1]. Therefore, PC requires a multidisciplinary approach involving generalist and specialist doctors, nurses and Allied Health and Social Care Professionals (AHSCPs). AHSCPs (such as Occupational Therapists, Physiotherapists, Psychologists, Dietitians, Speech and Language Therapists and Social Workers) work in partnership with health and social care professionals across all healthcare settings [2, 3]. Early access to PC for those with non-curable cancer not only improves patient symptoms and quality of life [4, 5]; it also reduces informal carer stress and depression [6], reduces financial strain on families and the health system [7] and increases survival [8].

Unfortunately, on a global scale, there is insufficient access to PC with as little as 14% of the world’s population having their PC needs addressed [1]. A large proportion of those in need of PC (approximately 78% of adults) live in low- and middle-income countries such as Colombia [1]. Colombia has 48 million residents, 77% reside in cities, 7.1% in small municipal settlements (villages) and 16% in scattered rural areas [9]. Colombia’s healthcare system is based on universal health coverage through three kinds of health insurance depending on the individual´s profession and capacity to pay: public, private, and special insurance [10, 11]. This universal health coverage includes 97.8% of the population [12]. However, inequitable access to health services and the lack of PC integration is one of the greatest barriers [10, 12].

In 2014, Colombia ordered legislation regarding the comprehensive management of patients with terminal, chronic, degenerative, and irreversible diseases in any phase of the disease [13]. The right to Advance Directives Forms (Resolution 1052 of 2016) was regulated as well as access to opioid medication (Circular 022 of 2016), rights of patients requiring PC (Circular 023 of 2016), to receive comprehensive care (Resolution 285 of 2018) [13, 14], decriminalization of euthanasia as part of the right to die with dignity (Resolution 971 of 2021) [15] and as of 2022, medically assisted suicide was also depenalised, under specific circumstances [16].

Despite all these regulations, access to PC remains limited [12]. In Colombia, 69 to 83% of deceased adults with chronic illnesses required PC, and approximately 30% of those who needed PC, died without receiving it [13]. The offer of PC is mostly hospital-based or ambulatory (hospices do not exist and home-based palliative care services are very scarce) and are concentrated in the main cities, with a scarcity of PC provision in small towns and rural areas – where transportation time from a health service to a home may be prolonged – [15]. Approximately 250,000 adults need PC annually, but there are only 1.6 services per million inhabitants, which reflects the insufficient offer and coverage of PC [12].

The literature has highlighted several barriers to accessing PC, in addition to the aforementioned limited offer. One of these is the lack of training and availability of health care professionals in rural areas (40.8 in rural vs. 102 professionals per 10 thousand inhabitants in urban areas) [18]. In 2012, only three of the 57 medical schools included formal training in PC in their undergraduate curriculum [17]. Moreover, many PC teams in Colombia comprise only nurses and physicians with a lack of involvement of other types of AHSCPs who are crucial to providing palliative care [2], such as psychologists, physiotherapists, occupational therapists, speech-language therapists, and nutritionists [18].

AHSCPs can provide significant contributions to improving the quality of life, care and attention of patients requiring palliative care and their caregivers, including non-pharmacological treatment and management of unpleasant symptoms associated with cancer [2]. They provide crucial rehabilitation to improve functioning of daily life [19] and improve patient comfort via nutrition, physiotherapy, and occupational therapy to manage debilitating symptoms such as fatigue, dyspnoea and weight loss [20–23]. Psychologists and social workers also address patient and family psychosocial needs such as pre and post bereavement support, and facilitate administrative processes related to health care insurance [24–26], while core functions such as communicating and swallowing that impact on patient dignity and comfort can be supported by speech therapists [27].

However, despite their crucial roles, the perspectives of AHSCPs around barriers and facilitators to palliative care for patients with non-curable cancer have not been explored. Combined with the scarce evidence base on barriers to PC in low- and middle-income countries such as Colombia [1], this study aims to address this knowledge gap by drawing on the perspective of underrepresented AHSCPs in two cities of Colombia: one in a medium sized city in an otherwise rural area (Popayan) and one very urbanized area (Bogota).

## Methods

Two focus groups were conducted for the World Cafe. The World Cafe method optimizes group intelligence by encouraging open discussions of ideas and experiences among diverse expert stakeholders [28]. It involves small group expert discussion around predetermined questions as informed by the World Cafe aims. The group interaction is similar to that of a focus group, but there are by definition two groups, each of which has a moderator. At the end of a first round of conversations around the central topic, the moderators of each group cross over to the other group, where they facilitate “new input” in each group to iteratively discuss and build consensus answers to the central questions of the session [28].

### Setting, sample and recruitment

AHSCPs with experience in PC were invited through social networks (WhatsApp, Facebook or email) in two cities in Colombia (Bogota and Popayan) in April 2022. Non-probabilistic snowball sampling was employed in order to recruit a range of AHSCPs. There is no consensus about the sample size required for a World Cafe design, therefore we adhered to previous recommendations suggesting six to ten participants per focus group [29].

### Data collection

The focus group topic guide was developed iteratively by epidemiologists, physicians, psychologists, nurses, and a registered dietitian nutritionist, all of them investigators of the overarching larger project “Proyecto Colibrí”. Proyecto Colibri is a collaborative project aimed at understanding patients with cancer in Colombia, and their relative or caregiver’s medical decision making at the end of life [30]. Seven questions about barriers and facilitators to PC were agreed upon (Supplementary Materials 1).

Participants provided written informed consent (which included permission for focus group recordings and publication of anonymised quotes) and were then scheduled for a two-hour focus group in Bogota. Due to travel and plenary meeting restrictions during a small COVID-19 peak, the two-hour focus group in Popayan was conducted virtually using the platform Microsoft Teams. The focus groups were recorded via audio (face-to-face meetings) and audio-video (online meetings).

The focus groups included two rounds of parallel small group discussions, lasting around 45 min each. Participants in the focus group rotated between tables containing paper and pens to note their thoughts. Participants in the online focus group alternated between virtual rooms (using the Microsoft Teams breakout room function to replicate in-person discussion tables). The host stayed at one “table” and provided a summary of the previous focus group discussion to the next rotating group of participants facilitating the building of experiences and ideas from each group. Finally, a larger group discussion was held, focused on building a consensus on key points from the summaries of smaller group discussions [25]. The hosts (CVM) included a PhD student in Clinical Epidemiology with qualitative methods training who was known to participants in Bogota, but not Popayan, and two nurses (MEGN, AMME) with experience in PC and previous training in the World Cafe method who also hosted in Bogota and Popayan, respectively. MEGN was not previously known to any of the participants of Bogota. AMME knew some of the participants in Popayan.

### Data analysis

The focus groups were transcribed verbatim in Spanish and translated into English by CVM, who is bilingual. The translation was reviewed by EdV, and thematically analysed for patterns in the data to construct the main categories and themes [31] by one of the researchers (TM). These themes were subsequently assessed and discussed by two other members of the research team (CVM, EdV). The research team had the opportunity to modify or add themes until consensus was reached to improve rigour. Participants were also invited to comment on the accuracy of the results as a member check of the validity and trustworthiness of the findings [31].

### Ethical considerations

Formal research ethics approval was obtained from the Pontificia Universidad Javeriana, Bogota, Colombia (FM-CIE-0086-17, number 2016/53). The study was conducted in accordance with the Declaration of Helsinki [32] and Colombian Resolution 8430 of 1993 [33]. The participants completed an informed consent statement prior to commencement of the World Cafe.

## Results

One World Cafe event was organised in each city. The groups were attended by 18 participants: nurses [3], nurse assistants [2], psychologists [2], psycho-oncologist [1], speech therapists [6], occupational therapist [1], registered dietitian nutritionists [2], and a social worker [1]. In Bogota, the group was made up of nurses [2], speech therapists [4], registered dietitian nutritionists [2], and a psycho-oncologist [1], and in Popayan the group was made up of nurses [1], nurse assistants [2], psychologists [2], speech therapists [2], an occupational therapist [1], and a social worker [1]. Table 1 describes the demographic information of the participants.

**Table 1:** Demographic characteristics of participants

Profession (N:18)	Bogota n:9 (%)	Popayan n:9 (%)
Nurses (n:3)	2 (22.2)	1 (11.1)
Nurse assistants (n:2)		2 (22.2)
Psychologist (n:2)		2 (22.2)
Psycho-oncologist (n:1)	1 (11.1)	
Speech therapist (n:6)	4 (44.4)	2 (22.2)
Social worker (n:1)		1 (11.1)
Occupational therapist (n:1)		1 (11.1)
Registered dietitian nutritionist (n:2)	2 (22.2)	

Participant quotes are included throughout the results section in order to support and illustrate interpretations made about the data.

An important context to note is that the majority of participants had experience with PC during internships, work experience, or through a MSc in PC nursing, and as such had good knowledge and understanding of this approach to care.

“My experience with PC has been with cancer patients. From the beginning of their treatment, from when they break the news to the end of their treatment or the moment of death. So, it really goes hand in hand” [Nursing: Masters in Oncology Nursing. Bogota].

“My experience in this area has been super rewarding both professionally and personally. I have had the opportunity to learn a lot… most of all to be aware of the impact that the PC approach has on people with oncological or chronic diseases… the importance of having an interdisciplinary approach… PC that aims to improve the quality of life” [ Nursing: Master student in PC 3. Bogota].

Five key themes emerged from the data in relation to both facilitators and barriers to accessing PC:

1. Humanising care.
2. Normalising PC: Referral at point of diagnosis.
3. Misunderstandings related to PC.
4. Barriers within the health care system.
5. Geographical barriers.

### Humanising Care

All participants agreed that the benefits of PC outweighed any disadvantages for both patient and family, as it helped to humanise this aspect of health care.

“Especially the PC specialist, Doctor X. He was always a very human person, so he always talked to the family. That is, the important thing is giving quality of life to the person, as a human being. And I think that, if one can help people to have quality of life in their last moments, then it would seem very important to me that we come together and work together as a team to reach that purpose” [Speech-Language Therapist D. Bogota].

“It is important to treat the family nucleus, not just the patient because the disease will not only be borne by the patient, the economic expenses, but the family will also go through situations of hunger, anxiety, need, displacement, difficulties. It (PC) really must be something integrative and that the whole family benefits from” [Nutritionist 3. Bogota].

In fact, the only disadvantages related to PC reported were not regarding the participants perspectives of PC as a specialty, but rather in relation to the barriers they had experienced in relation to referring patients to PC, as discussed later in this paper.

### Normalising PC: referral at point of diagnosis

Most of the participants described the reaction of patients and relatives being referred to PC as an event of uncertainty, fear, and hopelessness.

“Generally, they take it badly, it is like when they are diagnosed with cancer – the patient collapses, the family too, it is very difficult to face the diagnosis, and the same thing happens when they are referred to PC” [Nursing: Master’s Degree in Oncology Nursing. Bogota].

“But it always is related with the idea that they (the doctors) are not going to do anything anymore, and I feel that it is the opposite, it is that we are going to try to find […] what we can do to maintain or facilitate or favour […] People think that it is the opposite, it is that they send you over there to tell you ´bye, they don’t have anything anymore´, that you’re going to die” [Speech-Language Therapist D. Bogota].

“The surprise, the uncertainty, and the fear of “why am I being sent to PC”?” [Nursing: Master of Oncology Nursing. Bogota].

However, participants reported that patients who have experience with PC integrated into their routine care services had positive experiences of PC which transformed their uncertainty, fear and hopelessness to relief and hope that their needs would be met.

“Now people understand what they are going to treat, that, at least the pain symptom, when it was only palliative (referring to the specialty names such as Pain and PC) was more difficult to understand” [Speech-Language Therapist 2. Bogota].

“They receive it with relief, clearly there is uncertainty, fear, and the question “- I’m going to die, right?” is always present (Sighs). But…, already with all the explanation, the patients receive it with more, with more encouragement than fear” [Speech Therapist 2. Bogota].

Although it was an uncommon opinion, only one professional in the rural area (Popayan) described referral to PC as an approach exclusively focused on the end of life.

“I know that PC applies in the last moments, eh, like in the last, let’s say instances, of the person’s life.“ [Nursing assistant B. Popayan].

However, the majority of participants believed that PC referral should be made at the early point of a patient’s cancer diagnosis, so that it is seen as a natural part of the patient’s cancer-care pathway as and when needed.

“I totally agree with the others. It (PC referral) has to be from the beginning and it has to be a constant accompaniment… As well as supporting the patient it is very necessary to support families, understand that PC (sighs) is not for the terminal, on the deathbed, but that it is from the beginning (snaps fingers) that it is a constant process of support.” [Nutritionist 3. Bogota].

Early referral is not only crucial for normalising PC, but also to provide care in all areas that cancer affects the person and their family, and avoid the fear and feelings of abandonment that the negative stigma caused by misunderstanding of PC can generate among patients.

“We agreed that PC should be included from the diagnosis… All those stereotypes are going to be knocked down” [Speech-Language Therapist D. Bogota].

Participants alluded to the importance of a public health approach to normalising conversations around death and dying, so that having an advance directive and planning ahead is seen as everyone’s business, not just those with terminal illness.

“This issue of advance directives seems key to me! Even without having a disease, even without having a diagnosis of disease, it must be something that as a community and as a culture we should be able to talk about, but we are still very afraid to talk about death… We need to normalise advance directives and plan ahead” [Psychologist J. Bogota].

### Misunderstandings related to PC

AHSCPs feel it is their responsibility to educate patients on what PC is as they had witnessed first-hand the huge change in attitude and sense of peace this understanding gave both patients and their families. Without this information, families could often represent a key barrier to palliative referral, creating a “conspiracy of silence” [Nurse (1) Popayan], around the non-curative nature of a patient’s condition, and resulting in what staff viewed as a violation of the patients´ right to information.

“And it is the responsibility, I believe, that the health personnel who refers you explains what PC is going to be about, that it is not an end-of-life process. Rather, it will help you with pain management, with symptom management, with certain events that occur during the course (of your cancer). So, I think we should be clear in explaining what it is for referral to PC and not simply leave that referral to the imagination of the family member and the patient.” [Speech Therapist (2) Bogota].

“There must be continuity of care, not only an appointment at the beginning and it is over and it is never seen again. There must also be promptness, if I refer the patient to PC and the first available appointment is three or six months later, the patient may feel a total abandonment, while if PC comes in right from the start and he [the patient] receives a good explanation of what PC is, that he is not necessarily going to die, that one is going to treat the pain, that it will improve his quality of life, then people can have a different response… Because otherwise patients can fall into many lies, into many myths” [Nutritionist (3) Bogota].

However, the importance of education was also recognised at the level of the AHSCPs in order to improve PC provision, especially at undergraduate level within health-related programs.

“Our training in undergraduate programs is sometimes very short, very limited, even in many, many institutions there is no palliative training” [Nurse 1. Popayan].

In the same line of reasoning, participants recognised that they, as AHSCPs, can only do so much to overcome myths and misunderstandings around PC. Without a public health approach, it is very difficult to address the historical misinformation on social media and in the public domain.

“Patients have more and more access to information and clearly they’re going to search for something, on Google: what is PC? There they see the definitions of PC, and it’s like end of life, look for images on Google and it’s people crying and sad people, so they say, no, this is serious, my family member is going to die, so I think it has the disadvantage that the name [of PC] is very stigmatised” [Speech-Language Therapist 2. Bogota].

### Barriers within the health care system

Participants also reported several barriers to PC referrals at the health system level.

The lack of time to educate patients on PC was cited as a major barrier to PC referrals.

“You do not convey in 20 minutes, really what is necessary, is a little more dedication and a little more time. But we were talking about how the health system really doesn’t allow it, even though it’s completely necessary.” [Nutritionist 3. Bogota].

“We live in a, in a time, of no time, right? Where they require productivity, where they tell you, you have to see so many patients per shift, you have to invoice so many patients per shift. So, I think that something we all agree on, is that we have to reformulate our health system” [Speech-Language Therapist 2. Bogota].

Participants also described a triangular model of suffering for the patient, their family and their health care provider caused by the administrative burden of the patients’ health insurance who lack knowledge around PC and the importance of comfort care during a terminal illness.

“Starting from what the patient has to face in these situations that cause so much stress… Even those administrative barriers, which I think it is more difficult to fight, because we can have the interdisciplinary team within the institution, but when the patient leaves, the suffering and the ordeal begins for these patients. We do not talk about a comprehensive PC in that case, because the patient will go out many times to, to suffer all these consequences of the poor administration at the level of the health insurance entities” [Psychologist 2. Popayan].

“With patients in the terminal phase it seems especially painful to me … when you don’t… You can’t prescribe them supplements because basically they [the insurance] won’t authorise it because of the patient’s situation, but it’s basically condemning them [the patients] to starve, especially when there is a tumour in the gastric tract… and to think that the family has to see this happening.” [Nutritionist 3. Bogota].

A medicalised curative-based model of health care training also appeared to pose a barrier to PC referrals.

“Many times, we discussed it in the group. We were trained, in my case I am a nutritionist, to prescribe diets, calculate nutritional eating requirements…” [Nutritionist (A) Bogota].

“To take care of life, in the case of us nurses to take care of life and not to take care of death” [Oncology Nurse Specialist (B) Bogota].

Furthermore, as gatekeepers for decision-making, physicians could pose a barrier to AHSCPs referrals to PC unless the doctor considers it pertinent.

“I don’t refer for a doctor to see the patient, I, on the contrary, respond to the referral that the doctor gives for the patient” [Speech-Language Therapist. Bogota].

“How to approach the patient, but it is always limited by a filter, which is the doctor” [Nutritionist C. Bogota].

### Geographical barriers

Professionals described geographical differences in PC referrals, with those in urban areas having greater access to PC services compared to those living in more rural areas.

“Let’s say in Bogota, where there is an interdisciplinary program and these patients are diagnosed have a great advantage […] just as they start their oncological management with chemotherapy, radiotherapy and all this, they start, at the same time, PC management. I mean having an appointment with a palliative doctor, an appointment with a palliative nurse, an appointment with physiotherapy, speech therapy, psychology, and social work” [Nurse: Masters student in PC 3. Popayan].

“In fact, with PC, it should be at all levels of care, not only those institutions of high complexity but even in rural areas” [Nurse 1. Popayan].

Another evident barrier appeared to be in relation to inadequate resources in rural areas and the community to provide consistency and equity of care across settings.

“Another disadvantage could be the lack of offer… That is, wanting to refer a patient, but that there is no access by the providers or by the health institutions” [Nurse: Masters student in PC 3. Popayan].

“Outpatient accompaniment is a very complicated issue” [Nutritionist A. Bogota].

“It is practically impossible I would say… People who go out for homecare… There is no longer a psychologist, who follows both the patient and his environment… On that side we are already with a great failure” [Psychologist C. Popayan].

This lack of rural and community/home-based PC provision also has implications on repeated hospital admissions.

“The administrative part of the EPS [health insurance companies]… The patient often re-enters [the hospital] for emergencies, because there is no delivery of pain medications [Social work G. Popayan].

## Discussion

This study identified multiple difficulties in effective and equitable access to PC for patients who need it, ranging from misconceptions, fears of PC among patients and families, lack of undergraduate PC education for professionals, lack of equitable access to PC in rural areas and in the community setting, and having to navigate a burdensome and complicated health insurance system that does not cover comprehensive PC provision [34]. AHSCPs also reported barriers in terms of gatekeeping, with final decisions around initiation or not of PC resting with physicians and specialists [35], and the perspectives of other health care professionals involved in the care process commonly excluded. Similar findings have been reported previously [36].

However, experiencing and observing the humanisation of care that underlies the ethos of PC, to address physical, psychological and spiritual needs of patients, and which views patients as human beings, that are more than their illness [37] was a key facilitator in conveying the benefits of this approach to care to AHSCPs. This suggests that experiential learning, which as its name suggests, involves learning about PC via direct experience (such as shadowing specialist PC providers), is important for building staff competence, understanding and appreciation of PC for improving patient and family outcomes [38].

Participants in this study reported benefits of early PC provision for the patient and whole family. However, the wider research shows that caregivers in Colombia rarely receive psychological support during a terminal illness, thus compromising their physical health and increasing their risk of depressive symptoms [39, 40]. On the other hand, timely PC provision for the family nucleus is associated with early detection and management of caregiver burden, grief management, and return to working life [39], highlighting the importance of early referral.

Whereas access to PC is reported to be associated with better quality of life, stress management, family involvement, and even higher life expectancy, particularly when patients are referred early in the disease process, previous research indicates that referral to PC often occurs late: on average 30 to 60 days before death or in the late course of the illness [34].

In 2019, three out of 10 Colombians died while waiting to receive PC [13]. Though most of the participating AHSCPs in this study, from both rural and urban areas, described the importance of receiving PC early, one participant from the rural area still believed that PC services are exclusively oriented towards end of life care.

Early referral is complex for physicians because they must consider the disease process, and possible remission, alongside the challenges of having PC conversations, which require adequate time that is not always available to busy physicians [34]. In previous studies, some physicians mentioned that they always want to avoid eliminating “hope” among their patients, and therefore they usually keep offering any other treatment option before referring to PC [36].

Furthermore family members can hamper conversations regarding PC between all those involved as they may misunderstand that PC means that their loved one is imminently dying [36]. AHSCPs in this study therefore emphasised the importance of explaining what PC is to patients and their families in order to demystify erroneous beliefs that pose barriers to PC conversations and referrals. A recent randomised controlled trial supported this finding in demonstrating that simple information or short educational video can do much to improve PC understanding among the public, which may in turn reduce patient and family level barriers to PC referrals [41].

The importance of education for AHSCPs was also raised in this study’s findings. Participants reported what they termed a “conspiracy of silence”. This concept refers to an agreement sometimes unspoken to withhold information about the patient’s health condition and disease progression which has also been reported in the wider literature [42]. This lack of communication between AHSCPs, patients and their families around PC could also be related to the limited PC education found in Colombia’s undergraduate medical, nursing and AHSCPs curriculum [13].

Previous studies suggest that many health professionals associate PC exclusively with pain management, or the end of life. Such perceptions cause a passive attitude and indifference to PC among AHSCPs which may generate anxiety, fear, confusion, anger, and unnecessary suffering in the patient and their relatives if PC referrals are not made or made too late [42]. Participants from Bogota reported that the use of social media by patients and relatives to find information regarding PC is common, but often results in erroneous impressions of PC, that it is only end of life care or even other related concepts like euthanasia. However, social media can also represent a tool to disseminate a positive perception of PC [43]. For example, social networks, such as twitter and Facebook have contributed to opening a more positive, open dialogue around PC among patients, relatives, and health professionals [44].

In addition, participants felt that some of the misconceptions that patients and their families have about PC are associated with inadequate communication with AHSCPs. Thus, AHSCPs require effective communication skills that focus on patient needs, preferences, and their individual history. In a similar approach, Busch et al. described important aspects that influence the relationship between AHSCPs and patients, including respect for patient’s dignity, uniqueness, individuality and humanity, empathy, holistic approach, respect for patient’s autonomy and patient involvement, and verbal and non-verbal communication [45].

The findings from this study highlighted geographical barriers that resulted in inequity of palliative care provision for patients with non-curable cancer in more rural areas of Colombia. This is a persistent problem despite public policies [12] and local guidelines recommending integration of PC delivered by multi-disciplinary teams that intervene from the point of diagnosis to assess and resolve patient and family needs [46]. PC programs are mainly located in the capital cities (i.e., Bogota, Antioquia, Valle del Cauca, and Atlántico) [17]. Although rural departments, such as Cauca have similar numbers of patients requiring PC, Cauca only has 1.8% of services compared to widespread availability in urban areas of Colombia and has no home care service provision [13].

In Colombia, the coverage of PC services varies from between 0 and 72% [47]. These differences in PC provision are associated with the socio-economic developments of the regions: less developed regions, including most rural areas, have a more limited offer of PC services [47]. According to the WHO, this constitutes one of the biggest gaps in PC provision [48].

Provision of professional PC care in remote areas is very difficult on a practical level too: geographical and road conditions mean that a distance of perhaps 20 km could imply 3 or more hours of travel time. This has huge resource implication as it is not viable to have professionals spend that much time travelling to deliver PC. Therefore, informal caregivers should be provided with the tools (educational and practical) to provide basic PC to their loved ones, so that less intensive professional support is required.

To compound this yet further, health professionals with training in PC are commonly located in highly specialised health centres, excluding those focused on primary health care and again centred in larger cities [47]. To tackle these barriers, the WHO promotes the updating and creation of public policies on PC, that include the integration of PC in all health systems and primary care, recognizing holistic care needs (physical, social, psychological, spiritual) [48].

Policies in Colombia also need to address the inequity of PC education across all regions, and across all specialities involved in PC provision. Although the specialty of Pain Medicine and PC exists in Colombia since 1998, only 9.1% of medical schools have an independent curriculum subject on PC, and the educational offer of PC is even lower in nursing (5.3%), and psychology (3.4%) training [49]. Of note, the social work curriculum presents the largest educational offer (13.3%) [49].

In 2012, only three of the 57 medical faculties in Colombia included PC in their undergraduate curriculum [17]. Ten universities offered postgraduate training in PC, but only one of these had a multi-disciplinary program, the others were exclusively for physicians, nurses, and psychologists [13]. According to the latest report (2021) on the Current Status of PC in Colombia for undergraduate medicine, only six of the 32 departments had specific training on PC [13]. In nursing, the offer covers 14 departments out of 32, concentrated in the main capitals of the country [13].

The academic offer for PC education and training is even more limited for AHSCPs [13] with only one PC program available for AHSCPs [13]. Again, this program is centralized and located mainly in capital cities such as Bogota (67%), Cundinamarca (8%), and Antioquia (25%) [13]. Participants in our study also highlighted this lack of access to PC education [13] which results in a lack of knowledge regarding chronic pain and the use of opioid medications among health professionals, administrative personnel, and patients or relatives [13].

### Limitations and strengths

The limitations of this study include the non-probabilistic snowball sampling approach which could limit the representativeness of the participants, and thus the findings. Additionally, the majority of AHSCPs that participated in this study had experience with PC during internships, work experience, or through a MSc in PC nursing, and as such had good knowledge and understanding of this approach to care. As the wider literature and findings from this study have shown, this is not typical of AHSCPs in Colombia which further impacts on the transferability of the findings. However, a strength of this study was the Word Cafe Method to establish a structured discussion with a wide range of AHSCPs in both rural (Popayan) and urban areas (Bogota) to ensure diversity of participants and settings. To the best of our knowledge, this is also the first study to gather the perspective of AHSCPs about referrals to PC in Colombia. Future studies are required to analyse perspectives of other populations, for instance, primary health care, patients and caregivers. Moreover, it might be relevant knowing the barriers of other relevant issues in the legal framework of Colombia, such as the implementation of euthanasia and the recent approval of assisted suicide.

## Conclusions

This study has provided valuable insight into the perspectives of AHSCPs in Colombia in relation to barriers pertaining to equitable access to PC which to date has been missing. The main barriers lie in the misconceptions of patients, caregivers, relatives, and even health and AHSCPs that PC equates to end of life care only. This can be explained by the lack of PC education for health and AHSCPs, and negative connotations surrounding PC in the public domain. Access to PC is further challenged by lack of equitable access to PC in rural areas and in the community setting, and having to navigate a burdensome and complicated health insurance system that does not cover comprehensive PC provision [34].

This is a call for action starting at (1) a societal level for a public health approach in Colombia to tackle misperception’s around PC, (2) at a higher education authority level to ensure integration of PC education pre and post graduate level for all health and AHSCPs, (3) health insurance system level to ensure comprehensive PC provision, (4) health and social care board level to ensure adequate staffing levels for PC provision in primary/community care and rural areas. It is only by taking a whole systems level approach such at this, that takes due cognizance of all parts of the problem, that real change can happen.

### Electronic supplementary material

Below is the link to the electronic supplementary material.

Supplementary Material 1

Supplementary Material 2

## Data Availability

The datasets used and/or analysed during the current study are available from the corresponding author on reasonable request.

## List of abbreviations

PC: Palliative Care
AHSCPs: Allied Health and Social Care Professionals
