# Supplementary material for: Barriers and facilitators to palliative care for patients with non-curable cancer in Colombia: perspectives of allied health and social care professionals
Source: BMC Palliat Care. 2023 Oct 6;22:149. doi: 10.1186/s12904-023-01267-5 (PMC10557296; doi:10.1186/s12904-023-01267-5)
Supplement: Supplementary file 1 — Supplementary Material 1 [file 12904_2023_1267_MOESM1_ESM.docx]

**Supplementary Materials**

**Table S1. World Cafe questions English and Spanish.**

| **World Cafe questions** | |
| --- | --- |
| **English** | **Spanish** |
| How has your experience with Palliative Care been? | ¿Cómo ha sido su experiencia con Cuidado Paliativo? |
| Under which circumstances should palliative care be initiated? | **¿**Bajo qué circunstancias se debería iniciar el Cuidado Paliativo? |
| What characteristics should a patient have in order for indicated palliative care? | ¿Qué características debería tener un paciente para que sea indicado el Cuidado Paliativo? |
| What are the advantages and disadvantages of referring a patient to Palliative Care? | ¿Cuáles son las ventajas y desventajas de remitir a un paciente a Cuidado Paliativo? |
| What should be considered when deciding to refer a patient to Palliative Care? | ¿Qué se debe tener en cuenta a la hora de tomar la decisión de remitir un paciente al servicio de Cuidado Paliativo? |
| What elements do you use to decide to refer a patient to Palliative Care? | ¿Qué elementos utiliza usted para decidir la remisión a Cuidado Paliativo de un paciente? |
| How do patients react to being referred to Palliative Care? | ¿Cómo reaccionan pacientes al ser remitidos a Cuidado Paliativo? |
