# Supplementary material for: Barriers and facilitators to palliative care for patients with non-curable cancer in Colombia: perspectives of allied health and social care professionals
Source: BMC Palliat Care. 2023 Oct 6;22:149. doi: 10.1186/s12904-023-01267-5 (PMC10557296; doi:10.1186/s12904-023-01267-5)
Supplement: Supplementary file 2 — Supplementary Material 2 [file 12904_2023_1267_MOESM2_ESM.docx]

**Supplementary Materials**

**Table S1. World Cafe questions**

1. How has your experience with Palliative Care been?
2. Under which circumstances should palliative care be initiated?
3. What characteristics should a patient have in order for indicated palliative care?
4. What are the advantages and disadvantages of referring a patient to Palliative Care?
5. What should be considered when deciding to refer a patient to Palliative Care?
6. What elements do you use to decide to refer a patient to Palliative Care?
7. How do patients react to being referred to Palliative Care?
